# Prevalence of oral mucosal lesions among elderly Indian patients: A retrospective study

**DOI:** 10.6026/973206300213401

**Published:** 2025-09-30

**Authors:** Kavita Sharma, Mudita Chaturvedi, Rashi Dubey, Sriram Choudary N., Prasanthi Bajana, Dheeraj Voulligonda, Heena Dixit Tiwari

**Affiliations:** 1 Consultant Oral and Maxillofacial Surgeon, Bhopal, Madhya Pradesh, India; 2 Department of Oral and Maxillofacial Pathology, Dental Research Cell, Dr. D. Y. Patil Dental College and Hospital, Dr. D. Y. Patil Vidyapeeth, Pimpri, Pune, Maharashtra, India; 3 Department of Dental Surgery, S N Medical College, Agra, Uttar Pradesh, India; 4 Department of Oral & Maxillofacial Surgery, Dhanalakshmi Srinivasan Dental College & Hospital, Siruvachur, Perambalur, Tamil Nadu, India; 5 Department of Oral Medicine and Radiology, Government Dental College and Hospital, Vijayawada, Andhra Pradesh, India; 6 Department of Oral Medicine and Radiology, Mamata Institute of Dental Sciences, Hyderabad, Telangana, India

**Keywords:** Oral mucosal lesions, aged, India, prevalence, denture stomatitis

## Abstract

Oral mucosal lesions (OMLs) are common in elderly populations and often linked to risk factors such as tobacco use and denture wearing. Hence, this retrospective study analyzed clinical records of 354 Indian patients aged 60 years and above to identify the prevalence and distribution of OMLs. The overall prevalence was 54.7%, with leukoplakia (19.5%), lichen planus (17.8%), malignancy (13.0%), and denture stomatitis (9.0%) being the most frequent findings. Tobacco use and denture wearing showed significant associations with lesion occurrence, whereas sex and systemic diseases were not related. These findings highlight the considerable burden of OMLs among elderly Indian patients and emphasize the need for preventive care, habit modification, and improved denture hygiene to reduce disease risk.

## Background:

Oral mucosal lesions (OMLs) represent a significant burden in the elderly, who often present with multiple systemic illnesses and declining oral health resilience [1]. Studies across India have highlighted that more than half of older adults suffer from at least one mucosal lesion, demonstrating the magnitude of the problem [2]. Commonly identified lesions include leukoplakia, oral lichen planus and denture-related stomatitis, conditions that not only impair quality of life but also predispose to malignant transformation [3]. International epidemiologic investigations similarly report prevalence rates nearing 60% in geriatric cohorts, emphasizing that this is not a localized phenomenon but a global concern [4]. The Indian demographic, however, is unique owing to widespread betel quid chewing, tobacco use and inadequate denture hygiene, all of which exacerbate lesion occurrence [5]. Further, hospital-based analyses have shown higher lesion frequency in elderly males, likely due to cumulative exposure to deleterious oral habits [6]. Pathophysiologically, aging mucosa undergoes epithelial atrophy, reduced salivary flow and impaired tissue repair, increasing susceptibility to traumatic and inflammatory lesions [7]. Cross-sectional research has suggested that denture wearers have significantly higher odds of developing mucosal pathology compared with non-users [8]. Moreover, potentially malignant disorders such as leukoplakia and lichen planus are increasingly being recognized in older populations, necessitating vigilant surveillance [9]. Despite growing literature, few Indian retrospective analyses have specifically targeted elderly populations, leaving an evidence gap for hospital-based dental cohorts [10]. Therefore, it is of interest to retrospectively evaluate the prevalence, lesion types and risk associations of OMLs in elderly Indian patients, contributing to evidence-based geriatric oral health planning.

## Materials and Methods:

This retrospective study was conducted at a tertiary dental college hospital. Ethical approval was obtained from the Institutional Review Board. Clinical records of patients aged ≥60 years who attended outpatient services between January 2022 and December 2024 were reviewed. Records with incomplete demographic, diagnostic, or clinical details were excluded. Variables extracted included age, sex, systemic conditions (diabetes, hypertension), oral habits (smoking, betel quid and alcohol), denture wearing and diagnosis of oral mucosal lesions. Lesions were categorized according to WHO criteria: leukoplakia, oral lichen planus, malignancy, denture stomatitis and others (e.g., ulcers, oral submucous fibrosis). Data were entered into anonymized spreadsheets. Descriptive statistics (mean, SD, percentages) were calculated. Associations between OMLs and risk factors were tested using chi-square tests. A p-value <0.05 was considered statistically significant. Analyses were performed using SPSS v25. Post-hoc power analysis confirmed >80% power to detect a prevalence difference of 10%.

## Results & Discussion:

A total of 354 elderly patient records were included (202 males, 152 females; mean age 68.9 ± 6.3 years). Overall, 194 (54.7%) had one or more OMLs (Table 1 - see PDF & 2 - see PDF). The prevalence of 54.7% observed in this study reinforces prior Indian retrospective evidence, which reported similar rates among geriatric dental patients [11]. Comparable figures have been documented in large international surveys, where nearly 60% of elderly individuals exhibited one or more mucosal lesions [12]. The predominance of leukoplakia and lichen planus in our sample echoes previous epidemiological patterns, particularly in populations with high exposure to tobacco and betel quid [13]. Malignancies accounted for 13% of all lesions, aligning with reports that geriatric cohorts contribute substantially to the burden of oral cancer [14]. This underscores the urgent need for early detection and preventive strategies within routine geriatric dental care. The significant association between tobacco use and OMLs in our findings is consistent with established data linking smoking and chewing habits to potentially malignant disorders [15]. Similarly, denture-related stomatitis was strongly associated with prosthetic use, paralleling studies showing that poor hygiene and prolonged denture wearing significantly increase lesion risk [16]. Interestingly, our data showed declining lesion prevalence in those over 80 years. This may reflect survival bias, whereby individuals with high-risk habits and lesions are underrepresented in the oldest age groups, a phenomenon supported by geriatric epidemiologic studies [17]. While systemic diseases such as diabetes and hypertension were common in our cohort, no significant association with OMLs was found which is in agreement with other hospital-based studies suggesting local risk factors dominate lesion development [18]. From a clinical perspective, these results emphasize the need for integrating oral mucosal screening into routine geriatric dental visits. Preventive strategies should prioritize habit cessation programs, regular prosthesis maintenance and early biopsy of suspicious lesions. Comparable approaches have been successful in reducing oral cancer incidence in other high-risk populations [19]. Finally, despite the retrospective design, the congruence of our results with multicenter and global data underscores their validity and relevance. Future longitudinal studies are warranted to explore lesion progression, malignant transformation and the impact of preventive interventions in elderly Indian cohorts [20].

## Conclusion:

A large number of elderly Indian dental patients present with oral mucosal lesions, chiefly leukoplakia, lichen planus, malignancy and denture stomatitis. Hence, preventive care, habit intervention and prosthesis hygiene are essential for reducing this burden.
